# Characteristics, Management, and Outcomes of Acute Coronary Syndrome Patients with Cancer

**DOI:** 10.3390/jcm9113642

**Published:** 2020-11-12

**Authors:** Valentina Milazzo, Nicola Cosentino, Jeness Campodonico, Claudia Lucci, Daniela Cardinale, Carlo M. Cipolla, Giancarlo Marenzi

**Affiliations:** 1 Centro Cardiologico Monzino IRCCS, 20138 Milan, Italy; valentina.milazzo@ccfm.it (V.M.); nicola.cosentino@ccfm.it (N.C.); jeness.campodonico@ccfm.it (J.C.); claudia.lucci@ccfm.it (C.L.); 2 Cardioncology Unit, European Institute of Oncology, IRCCS, 20141 Milan, Italy; daniela.cardinale@ieo.it; 3 Cardiology Division, European Institute of Oncology, IRCCS, 20141 Milan, Italy; carlo.cipolla@ieo.it

**Keywords:** acute coronary syndrome, cancer, chemotherapy, prognosis

## Abstract

Patients with cancer are at increased risk of cardiovascular disease, with a reported prevalence of acute coronary syndrome (ACS) ranging from 3% to 17%. The increased risk of ACS in these patients seems to be due to the complex interaction of shared cardiovascular risk factors, cancer type and stage, and chemotherapeutic and radiotherapy regimens. The management of ACS in patients with cancer is a clinical challenge, particularly due to cancer’s unique pathophysiology, which makes it difficult to balance thrombotic and bleeding risks in this specific patient population. In addition, patients with cancer have largely been excluded from ACS trials. Hence, an evidence-based treatment for ACS in this group of patients is unknown and only a limited proportion of them is treated with antiplatelets or invasive revascularization, despite initial reports suggesting their beneficial prognostic effects in cancer patients. Finally, cancer patients experiencing ACS are also at higher risk of in-hospital and long-term mortality as compared to non-cancer patients. In this review, we will provide an overview on the available evidence of the relationship between ACS and cancer, in terms of clinical manifestations, possible underlying mechanisms, and therapeutic and prognostic implications.

## 1. Introduction

Patients with cancer are at increased risk of cardiovascular disease [1]. This is because of the high prevalence of both diseases, the higher average age of cancer patients, and the presence of common risk factors, such as smoking [2]. Moreover, some cancers, as well as some of their therapies, should be themselves considered true cardiovascular risk factors. For these reasons, the occurrence of acute coronary syndrome (ACS) in cancer patients is not uncommon [3,4]. Notably, ACS may develop before or after establishing a diagnosis of cancer; sometimes, the diagnosis is made during hospitalization for ACS and, conversely, in some cases, ACS may occur during hospitalization for active cancer [5]. The coexistence of ACS and cancer often complicates treatment because the therapy of one disease can adversely affect the prognosis of the other [6]. In addition, guidelines for the treatment of ACS are based on studies that have excluded patients with cancer and may, therefore, not be suitable for neoplastic patients. Thus, the management of cancer patients developing ACS is not supported by clear evidence, and it represents a challenge that should be addressed by cardiologists in close collaboration with oncologists in a multidisciplinary, patient-centered approach [7]. The aim should be to customize the treatment for each patient based not only on the type of ACS (unstable angina vs. non-ST-segment myocardial infarction (NSTEMI) vs. ST-segment myocardial infarction (STEMI)), but also of the type of cancer, past and current cancer therapies, future therapeutic programs, and in relation to the possible different prognoses of the two diseases. Due to the continuous improvement in the survival of cancer patients, their cardiovascular care has received increasing attention, and demands in this area have and will continue to grow considerably [7].

The purpose herein is to provide an overview on the available evidence of the relationship between ACS and cancer, in terms of clinical manifestations, possible underlying mechanisms, and therapeutic and prognostic implications.

## 2. Prevalence and Clinical Characteristics of ACS in Cancer Patients

**Figure 1:**
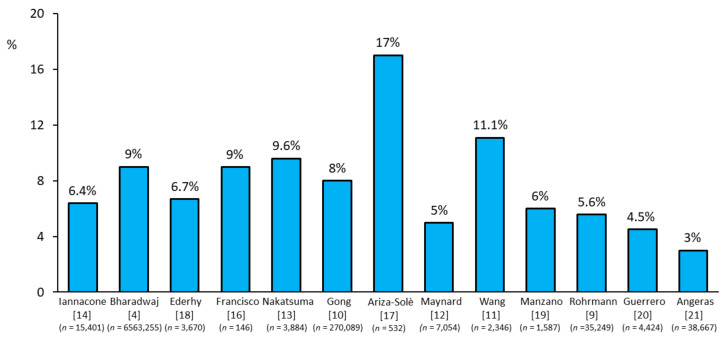
Prevalence of acute coronary syndrome events reported in cancer patients across studies [4,9,10,11,12,13,14,16,17,18,19,20,21].

Cancer disease has been reported to be present in up to 17% of patients with ACS [3,4,7,8]. Very recent data from 6.5 million patients presenting with an acute myocardial infarction (AMI) between 2004 and 2014, included in the US National Inpatient Sample (NIS) database, showed a prevalence of 9% of patients with cancer [3]. This figure was composed of 2.8% of patients with current cancer and 6.8% with a historical diagnosis of cancer. Of note, prostate, breast, colon, and lung cancers were the four most common types of cancer associated with AMI. Other ACS registries confirmed these findings, with a reported prevalence of cancer among ACS patients ranging from 3% to 17% [9,10,11,12,13,14,15,16,17,18,19,20,21] (Figure 1).

However, the true prevalence of cancer among ACS patients is difficult to estimate because many registries did not include this information among the variables collected and, when they did, patients with active cancer and those with a previous, even old, history of cancer were usually pooled together. When only ACS occurring during hospitalization for active cancer is considered, its incidence is lower. A retrospective analysis on 25,165 medical records of patients hospitalized to a Polish oncological center for cancer treatment reported an occurrence of ACS in 0.14% of patients [22]. Another retrospective analysis by Park et al. [23], who screened 5300 patients with active hematologic malignancies, identified 73 cases (1.4%) of ACS (unstable angina in 8%, NSTEMI in 78% and STEMI in 14% of cases) during hospitalization for cancer. Although the incidence of ACS in patients hospitalized in oncological units with active cancer seems to be low, this represents a very high-risk group, characterized by in-hospital mortality rates of about 25% [22,23].

The most frequent type of ACS in cancer patients is NSTEMI, but its presentation is often different from that observed in the general population [24]. The prevalence of silent ischemia is higher in cancer patients. The most common symptom is dyspnea, followed by chest pain, hypotension, and heart failure [24]. Frequently, chest pain is masked by analgesics prescribed to treat cancer pain or by the neurotoxic effects of chemotherapy [25]. The Bleeding complications in a Multicenter registry of patients discharged after an Acute Coronary Syndrome (BleeMACS) project reported that ACS patients with a concomitant cancer are older, with multiple comorbidities, and that they experience an NSTEMI more frequently [14]. Furthermore, in a population of 201 patients with active cancer and ACS, 76% of them presented with type 1 AMI while 24% presented with type 2 AMI [24]. No significant differences were observed between the two groups regarding demographics, risk factors, history, and cancer type. However, patients with type 2 AMI had a more advanced cancer and were more frequently receiving active chemotherapy. Moreover, they often had lower platelet counts and anemia [24]. Notably, in ACS patients, both thrombocytopenia and anemia have been demonstrated to be associated with a higher bleeding risk and with a poorer prognosis, independently of the presence of cancer [26,27].

## 3. Mechanisms of ACS in Cancer Patients

The pathophysiology of ACS in cancer patients is complex, and the ACS risk seems to depend not only on the traditional cardiovascular risk factors but also on cancer type, stage, treatment, and on cancer-associated hypercoagulability. In particular, ACS may be induced by cancer treatment (i.e., chemotherapy, radiotherapy, or surgical treatment) (Table 1).

Arterial thrombotic events, including ACS, are common in patients with cancer. Navi et al. [28] found, in 279,719 pairs of patients with cancer and matched control patients included in a retrospective study, a 6-month cumulative incidence of AMI of 2.0% in patients with cancer compared with 0.7% in control patients (hazard ratio (HR) 2.9; 95% confidence interval (CI) 2.8–3.1). Excess AMI risk varied by cancer type, with lung, gastric, and pancreatic cancers presenting the highest risk, and correlated with cancer stage [28]. Cancer can induce a pro-thrombotic state through alteration in platelet activity, coagulation and fibrinolytic systems, and endothelial function. Indeed, tumor cells have the ability to induce platelet activation and aggregation in-vitro [29]. Moreover, arterial thrombotic events increase in patients receiving chemotherapy [6,7]. In particular, endothelial dysfunction, increased expression of pro-inflammatory cytokines, oxidative stress, alteration of coagulation, and platelet activity are the most common mechanisms of chemotherapy-related arterial thrombotic events in cancer patients [30].

Cancer therapy can also increase the risk of ACS by promoting the development of cardiovascular risk factors, such as metabolic syndrome, hypertension, hyperglycemia, and hypertriglyceridemia [31] or causing direct vascular or myocardial injury. For instance, androgen suppression therapy, used for the treatment of prostate cancer, is associated with an increased incidence of metabolic syndrome, and hence AMI [32].

**Figure 2:**
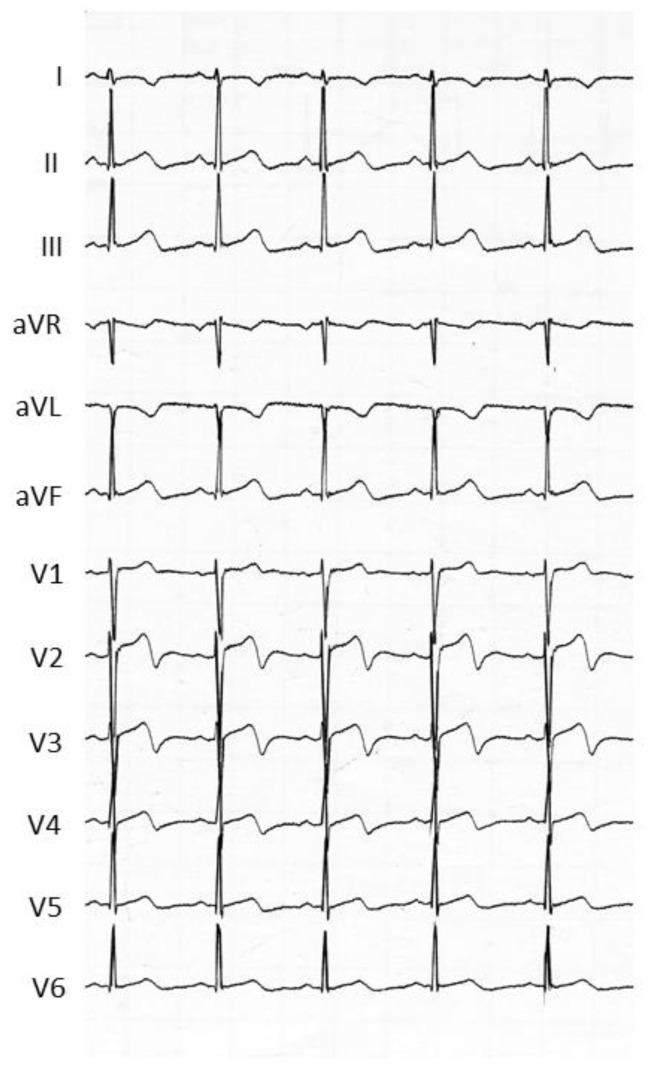
Acute electrocardiographic changes associated with typical chest pain observed in a cancer patient treated with 5-fluorouracil. In this patient, the coronary angiography was normal.

Fluoropyrimidines, which include 5-fluorouracil (5-FU) and capecitabine, form the cornerstone of several different chemotherapy regimens. Indeed, 5-FU is the third most commonly used chemotherapeutic agent in the treatment of solid malignancies across the world. At the same time, fluoropyrimidines are the second most common drugs associated with cardiotoxicity after anthracyclines [33], with a reported incidence of 2–34% for 5-FU and of 3–9% for capecitabine [34,35]. The most common manifestation of cardiotoxicity associated with fluoropyrimidines is chest pain, presenting as atypical chest pain, angina on exertion or at rest, and ACS [36,37]. The mean reported time interval between 5-FU administration and the onset of cardiac symptoms is three days (range two to five days). Symptoms are usually relieved within 48 h of drug discontinuation, but they frequently recur when infusion is restarted [37]. In parallel, the associated electrocardiographic abnormalities (acute ST-segment changes and T wave inversion (Figure 2)) are mostly resolved from within a few hours to up to three days of withdrawal [37].

Patients with cardiovascular disease are at higher risk of cardiotoxicity [38]. Moreover, infusional regimen of 5-FU is associated with a greater risk of cardiotoxicity than bolus regimens. The mechanisms underlying 5-FU cardiotoxicity are not fully understood, but coronary vasospasm and thrombus formation are the most widely accepted hypotheses [36,37,38]. This agent can also cause direct cellular damage of both cardiomyocytes and endothelial cells [36,37,38].

A cisplatin-based treatment regimen is also implicated in developing arterial thrombosis and ACS in cancer patients through endothelial cell damage and increased von Willebrand factor activity. Cancer patients treated with cisplatin have a 6–10% risk of developing venous or arterial thrombosis, including a 2% rate of ACS and cerebrovascular events, mostly occurring within the first 100 days (median 48 days) of starting cisplatin [39].

Other anticancer agents have been associated with an increased ACS risk. Vascular endothelial growth factor inhibitors (bevacizumab, sorafenib, sunitinib) may induce cardiac ischemia and arterial thrombosis [40]. The incidence of angina ranges from 1% to 15% of patients treated with these agents, and it seems to be due to an alteration of the activity of endothelial nitric oxide synthase and to increased oxidative stress [41]. Interestingly, accelerated atherosclerosis has been observed in patients receiving treatment with sorafenib, progressing from a normal coronary angiogram to critical stenosis of the left main over the course of only 4 years in a patient treated for metastatic renal cell carcinoma [42]. The use of immunomodulatory agents, especially lenalidomide and pomalidomide, and of the proteasome inhibitor carfilzomib is also associated with an increase in cardiovascular events [43]. Immunomodulatory agents are known to increase the risk of thrombotic events and AMI [43]; however, the mechanisms underlying this association are still not well known. Carfilzomib, currently approved for treatment of relapsed and/or refractory multiple myeloma, is also associated with an increased ACS risk [44]. In particular, a safety analysis of phase 2 clinical trials reported a 3% incidence of ACS, with most of these events occurring soon after the first cycle of chemotherapy [45]. Finally, although the most severe cardiac complication of the treatment with immune checkpoint inhibitors is myocarditis, new data show that use of these molecules can be also associated with ACS [46]. The activation of immune cells in coronary atherosclerotic plaques seems to contribute to the destabilization of the atherosclerotic lesions, leading to plaque rupture and ACS [47].

Finally, it should be highlighted that some chemotherapy drugs, such as anthracyclines, trastuzumab, and immune checkpoint inhibitors, can induce myocardial injury through mechanisms not dependent on myocardial ischemia [48,49,50]. The finding of increased troponin values in patients treated with these drugs may lead to suspicion of ACS. However, in most cases, the troponin increase does not show the typical dynamic rise and/or fall pattern of ACS, and it is not associated with clinical evidence of acute myocardial ischemia as reflected by symptoms, new ischemic electrocardiographic changes, and new regional wall motion abnormalities at echocardiogram. In the absence of these additional clinical elements, further diagnostic investigation with coronary angiography is not justified [51,52]. Although of non-ischemic origin, the myocardial injury associated with the use of these agents should not be underestimated because of its important therapeutic and prognostic implications [53].

Radiotherapy may be associated with a higher incidence of ACS, through the development of atherosclerotic disease complicated by plaque rupture and thrombosis, and potentially with coronary spasm [48,54]. Coronary ostia and proximal segments are typically involved and the most exposed coronaries are the left anterior descending during left breast irradiation and the left main stem, circumflex, and right coronary arteries during treatment for Hodgkin lymphoma [55]. Although the cardiac consequences may be rapid, with ACS or sudden death as initial manifestations, radiotherapy-induced coronary artery disease is more often asymptomatic for a long time, with clinical manifestation typically occurring 15–20 years after the initial treatment [7]. Notably, patients younger than 50 years tend to develop coronary artery disease in the first decade after treatment, while older patients have longer latency periods. The risk of developing ACS after chest irradiation is higher in patients treated with concomitant anthracyclines, in young patients, and in those with known cardiovascular risk factors or established coronary artery disease [7].

## 4. Clinical Management of ACS in Cancer Patients

The management of patients with ACS and active cancer is particularly difficult and challenging because they have systematically been excluded from prospective studies and trials assessing the efficacy and safety of ACS treatment. As a result, their treatment is not supported by clear recommendations, and current guidelines for an invasive and conservative treatment of ACS cannot be easily applied to all patients with cancer. There is growing awareness that cancer treatment has negative effects on the optimal management of ACS. Indeed, on the one hand, uncertainty in the ACS diagnosis increases as many chemotherapy agents can induce an increase in plasma troponin levels [56]. On the other hand, the use of potent antithrombotic therapies, the mainstay of ACS treatment, exposes these patients, who are more susceptible to bleeding, to an increased risk of hemorrhagic complications [57]. The combination of these factors makes the optimal treatment for these patients uncertain, with many concerns about its effectiveness and safety, and contributes to explain the lower survival rate reported in these patients [58,59].

**Figure 3:**
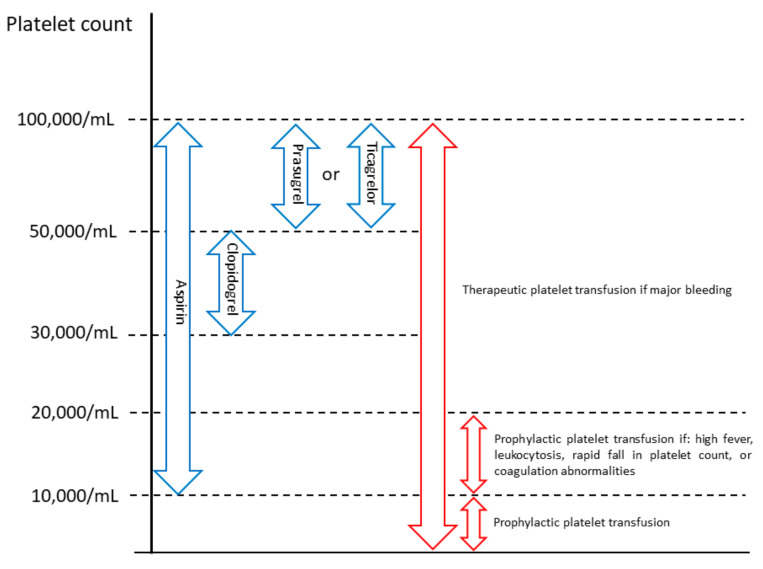
Management of antiplatelet therapy in cancer patients with acute coronary syndrome and thrombocytopenia (platelet count <100,000/mL) and no active bleeding, suggested by the Society for Cardiovascular Angiography and Interventions [62].

Observational data showed that ACS patients with cancer are less likely to receive guideline-recommended medications for ACS, with optimal medical therapy being prescribed in only one-third of them [59], despite the reported reduction in serious cardiovascular events by 30% during a 12-month follow-up in patients with ACS and cancer undergoing percutaneous coronary intervention and receiving optimal medical therapy [14]. Yusuf et al. [58] retrospectively analyzed 456 AMI patients with active cancer, including 70 patients who presented with STEMI; only 211 (46%) of them received aspirin. One-year survival was higher in patients treated with aspirin than in those without (34% vs. 18%). After adjustment for demographic baseline differences, aspirin use was significantly associated with improved survival [58]. The main reason why cancer patients are not treated with aspirin is the frequent concomitant thrombocytopenia [6]. About 10% to 25% of cancer patients have thrombocytopenia, defined as a platelet count of less than 100,000/mL [60]. However, a retrospective analysis by Sarkiss et al. [61] showed that among cancer patients with ACS and thrombocytopenia (median platelet count 32,000/mL), those who did not receive aspirin had a 7-day survival rate of 6% compared to 90% in those who received aspirin. Although bleeding occurred more frequently in ACS patients with thrombocytopenia than in those without, in patients with thrombocytopenia it did not differ significantly according to aspirin use (18% vs. 15%) [58]. In line with the results of this study, the current expert consensus statement on the management of cardio-oncology patients in the cardiac catheterization laboratory, by the Society for Cardiovascular Angiography and Interventions (SCAI), suggests aspirin administration unless platelet count is less than 10,000/mL [62] (Figure 3).

Similar data have been reported on the use of statins. In the study by Yusuf et al. [58], only 21% of cancer patients with acute myocardial infarction were prescribed statin therapy at hospital discharge. Moreover, a recent retrospective German study by Mrotzek et al. [63], comparing patients with and without cancer undergoing coronary angiography, found that statin therapy was significantly less frequent in the cancer group (29% vs. 49%), although there were no differences regarding patients’ medical history of known coronary artery disease and cardiovascular risk factors between cancer and control groups. It can be speculated that contraindications, concomitant comorbidities, cancer staging and complications of cancer therapy may explain the undertreatment of these patients. However, the beneficial effects of lipid-lowering treatment observed in ACS patients seem to also be maintained in cancer patients [59].

The same considerations apply to the use of myocardial revascularization in cancer patients presenting with ACS. Indeed, the use of an early invasive strategy consisting of percutaneous coronary intervention is also associated with improved outcome after ACS in cancer patients [14,64]. A study comprising 49,515 ACS patients with cancer found that the in-hospital mortality rate of patients undergoing myocardial revascularization was significantly lower than that of patients receiving conservative medical therapy [65]. Despite this growing evidence, a less frequent use of percutaneous coronary intervention with drug-eluting stents in patients with a history of cancer admitted for ACS has been consistently reported [9,15,58]. A study performed at the MD Anderson Cancer Center (Houston, Texas) found that, among cancer patients with AMI, only 3% underwent percutaneous coronary intervention and only 6% of STEMI patients underwent primary angioplasty [58]. Similarly, Pothineni et al. [15] analyzed the United States National Inpatient Sample and found that the utilization of primary angioplasty in STEMI patients with cancer varied according to the type of cancer, ranging from 17% in patients with colon cancer to 31% in those with breast cancer. Notably, cancer patients treated with primary angioplasty were less likely to die than those who did not receive myocardial revascularization [15]. Not only are patients with active cancer and ACS less frequently referred to the catheterization laboratory, but also those with a previous history of cancer; in the National Registry of Acute Myocardial Infarction in Switzerland (AMIS Plus), ACS patients with a history of cancer were less likely to undergo percutaneous coronary intervention than those without (68% vs. 73%) [9]. The presence of cancer may limit the use of cardiac catheterization because of frailty caused by aggressive chemotherapy treatments in these patients. Similarly, thrombocytopenia secondary to myelo-suppressive chemotherapy or to hematologic malignancies could explain the decreased use of coronary stents due to the need to limit the duration of dual antiplatelet therapy. Notably, when percutaneous coronary intervention is considered as a treatment option for cancer patients presenting with ACS, a higher risk of stent thrombosis should be taken into account. Several registries have demonstrated an underlying hypercoagulable state that predisposes cancer patients to a higher risk of stent thrombosis. The Dutch Stent Thrombosis Registry found that, among 437 patients diagnosed with definite stent thrombosis, 10% had active cancer [66]. In daily clinical practice, the use of bare-metal stents, requiring a shorter duration of dual antiplatelet therapy, is often preferred in cancer patients scheduled for surgical or chemotherapy treatment in the short term after ACS. However, a retrospective chart review of patients treated with bare-metal stents at a single center in Germany reported a higher rate of in-stent thrombosis in patients with cancer than in those without (5.6% vs. 0.8%) [67]. Possibly, this concern could be overcome by the use of new-generation stents in these patients requiring a short-duration single or dual antiplatelet therapy.

**Figure 4:**
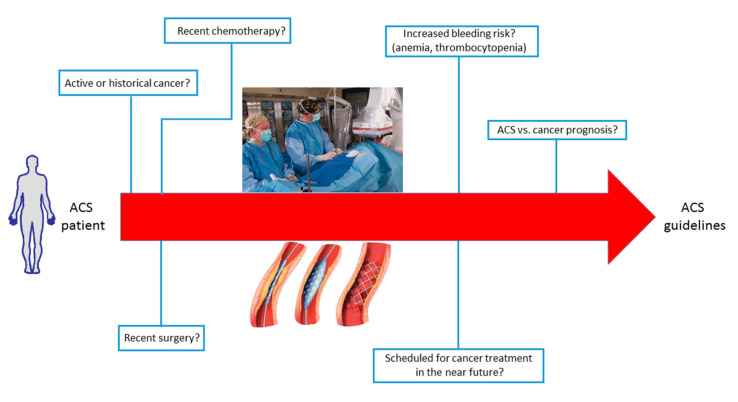
Schematic illustration of the possible issues and clinical challenges to consider in the management of acute coronary syndrome (ACS) patients with cancer.

In summary, a tailored approach appears important to reduce on the one hand the risk of in-hospital cardiac death during ACS and, on the other hand, to allow cancer patients to receive the best treatment for their underlying disease. A schematic illustration of the possible issues to consider in the management of ACS patients with cancer is shown in Figure 4.

## 5. Outcomes of Cancer Patients with ACS

While the prevalence of cancer patients in the setting of ACS has been investigated more, representing a growing, challenging population, there are limited data that sufficiently address their outcome. To date, there are only few observational, mainly retrospective, studies comparing the clinical outcomes of patients with and without active or historical cancer and presenting with ACS. These analyses consistently reported that such patients are at higher risk of in-hospital and long-term mortality than those without cancer [13,15,56,65] (Table 2).

A very recent analysis on a large AMI population revealed that patients with current cancer have a 50% increased risk of major in-hospital adverse cardiac events than those without cancer [4]. Conversely, AMI patients with historical cancer were at no increased risk of adverse outcomes. In particular, in-hospital mortality was 11% in patients with active cancer and 5.4% and 5.7% in patients with and without a prior history of cancer, respectively. A similar pattern was reported for bleeding complications, where the active cancer group had twice the rates of bleeding than the historical cancer and no cancer groups (18%, 10%, and 9%, respectively). Moreover, lung cancer was associated with the highest in-hospital mortality (odds ratio (OR) 2.7) and major adverse cardiovascular and cerebrovascular complications (OR 2.4), while colon cancer was associated with the highest risk of bleeding (OR 2.8). Irrespective of the type of cancer, the presence of metastases was associated with worse in-hospital outcomes [4].

In another study by Pothineni et al. [15], focusing on about 3.8 million STEMI patients (1.5% of whom had active cancer), patients with cancer had a significantly higher in-hospital and 1-year mortality than those without a history of cancer (in-hospital 9.1% vs. 3.4%; 1-year 17.4% vs. 6.5%). The presence of cancer, diagnosed in the 6 months preceding the acute cardiac event, was an independent risk factor for increased 1-year mortality (adjusted HR 3.3). In the BleeMACS project, the rate of a composite endpoint of all-cause death, AMI, and bleeding events, and of bleeding alone at 1-year follow-up was significantly higher in ACS patients with cancer than in non-cancer patients (composite endpoint 15.2% vs. 5.3%; bleeding 6.5% vs. 3.0%) [14]. In the study by Yusuf et al. [59], mortality in cancer patients was high, with a 1-year survival rate after AMI of only 26%. Patients with advanced cancer were twice as likely to die, and previous chemotherapy and chest radiotherapy were strongly associated with increased 1-year mortality risk [53]. Finally, a recent meta-analysis confirmed the worse in-hospital and 1-year prognosis of ACS patients with cancer [67]. Even if the magnitude of the relative risk of early and late all-cause mortality should be tempered by the heterogeneity among studies considered in this study, all reports included consistently showed a worse prognosis in such patients [68]. The Coronary Revascularization Demonstrating Outcome Study in Kyoto (CREDO-Kyoto) Registry Cohort-2 (12,180 patients, 27% of whom had ACS; history of cancer 9.1%) found that the cumulative 5-year incidence of cardiovascular death was significantly higher in patients with cancer (12.4% vs. 7.5%) [13]. Again, their excess risk of cardiovascular death remained significant after adjustment for major confounders. Findings were similar for other cardiovascular-related outcomes, as for all-cause death, non-cardiac death, heart failure readmission, and major bleeding. Subgroup analysis in ACS patients only (*n* = 3309) demonstrated findings consistent with those of the main analysis [13]. Finally, an analysis from the Duke database (*n* = 15,008; ACS, 72%; history of cancer 3.3%) found that, after a 14-year follow-up, cardiovascular mortality was not different between groups (31.4% vs. 27.7%), while all-cause death was significantly higher in patients with a history of cancer than in those without (79.7% vs. 49.3%) [69]. This suggests that non-cardiovascular comorbidities may have a greater prognostic relevance over the years after ACS in cancer patients.

## 6. Future Perspectives

Future research in this field is warranted to elucidate how to improve management of ACS patients with cancer. Indeed, there are still unmet needs and gaps in knowledge to guide best practice. Since patients with cancer are usually excluded from randomized trials, the most critical source of scientific evidence derives from observational studies and registries. The details of cancer, such as active or history of cancer, the duration of the oncologic disease before index event, the type of cancer, clinical stage, and treatment for cancer, should be systematically collected in ACS registries. A greater availability of these data could allow to define the real prevalence of cancer in patients with ACS. Moreover, it would allow a better characterization of the clinical and prognostic relevance of ACS in relation to the different type and expected outcome of cancer, thus customizing patient treatment.

In cancer patients, another relevant challenge is their increased bleeding risk, which limits the use of antithrombotic therapies after ACS. Several risk scores, including the Dual AntiPlatelet Therapy (DAPT), the PREdicting bleeding Complications In patients undergoing Stent implantation and subsEquent DAPT (PRECISE-DAPT), and the Patterns of Non-Adherence to Anti-Platelet Regimen in Stented Patients (PARIS) scores, validated for the definition of the risk/benefit profile of short vs. prolonged dual antiplatelet therapy duration in patients undergoing percutaneous coronary intervention, did not include cancer [70]. Therefore, the best antithrombotic strategy and duration in cancer patients with ACS remain unclear and should be addressed by future studies. In particular, the recent achievements in coronary stent technologies, with thinner struts, an absent or bioresorbable polymer, and sharper imaging-assisted implantation techniques, have further reduced rates of stent thrombosis and restenosis, allowing a shorter dual antiplatelet therapy, as short as 1 month in high-bleeding risk patients, including those with cancer [71,72]. Indeed, they are the ones who could benefit most from these technological advances.

## 7. Conclusions

In conclusion, patients with a current or historical diagnosis of cancer who present with ACS have more comorbidities than those without cancer. The majority of them is treated conservatively and has worse outcomes, such as higher in-hospital and long-term mortality. When treating ACS patients with cancer, clinicians often face several clinical and therapeutic conundrums, given the lack of robust evidence from the literature. These patients should, however, be approached putting ACS in the context of the expected cardiac and oncologic prognosis and tailoring their treatment accordingly.

## Figures and Tables

**Table 1:** Cancer treatments more frequently associated with acute coronary syndromes and their established or proposed pathophysiological mechanisms.

Agent	Pathophysiological Mechanism
Fluoropyrimidines (5-fluorouracil, Capecitabine, Gemcitabine)	coronary vasospasm, thrombosis, endothelial injury
Cisplatin	pro-coagulant state, coronary thrombosis (endothelial damage, thromboxane production, platelet activation and aggregation)
Vascular endothelial growth factor inhibitors	endothelial dysfunction, coronary vasospasm, vascular remodeling, inflammation, platelet
(Bevacizumab, Sorafenib, Sunitinib)	activation interference with plaque neovessel formation and integrity, increased endothelin-1
	production, oxidative stress, accelerated atherosclerosis
Immunomodulatory agents (Lenalidomide, Pomalidomide)	arterial thrombosis
Proteasome inhibitors (Bortezomib, Carfizomib)	thrombosis?
Radiotherapy	Endothelial injury, plaque rupture, thrombosis, fibrosis of the vessel wall, accelerated atherosclerosis

**Table 2:** Characteristics of studies investigating the prognostic impact of cancer in patients with acute coronary syndrome.

First Author (Ref#)	Year of Publication	Study Design	ACS Type	Cancer Patients (*n*)	Follow-Up Length	Mortality Rate (%) vs. Controls	Mortality Risk vs. Controls OR/HR (95% CI)
Yusuf [58]	2012	Retrospective	AMI	456	1 year	74%	-
Wang [11]	2016	Retrospective	STEMI	261	5 years	In-hospital: 7.7% vs. 4.9% 5 years: 34.2% vs. 16.8%	- HR 2.46 (1.96–3.09)
Nakatsuma [13]	2018	Retrospective	CAD (29% AMI)	1109	5 years	33% vs. 15.2%	HR 1.80 (1.60–2.01) *
Rohrmann [9]	2018	Retrospective	STEMI NSTEMI	1981	In-hospital	10.7% vs. 7.6%	OR 1.45 (1.17–1.81)
Gong [10]	2018	Retrospective	AMI	22,907	Median 10 years	30 days: 13.6% vs. 12.3% 1 year: 23.4% vs. 20.4% 11 years: 54.8% vs. 49.0%	HR 1.12 (1.07–1.17) * HR 1.16 (1.12–1.20) * HR 1.21 (1.17–1.25) *
Iannacone [14]	2018	Retrospective	ACS	858	1 year	11.7% vs. 3.2%	HR 2.1 (1.8–2.5) *
Park [23]	2019	Retrospective	ACS	73	1 year	In-hospital: 21.9% 1 year: 58.9%	-
Bharadwaj [4]	2019	Retrospective	AMI	596,301	In-hospital	7.2% vs. 5.7%	-
Ederhy [18]	2019	Retrospective	AMI	246	5 years	In-hospital: 8.9% vs. 5.4% 5 years: 52.8% vs. 28.1%	OR 1.15 (0.68–1.94) * HR 1.36 (1.08–1.69) *
Styczkiewicz [22]	2020	Retrospective	ACS	36	1 year	67%	-

ACS = acute coronary syndrome; AMI = acute myocardial infarction; CAD = coronary artery disease; HR = hazard ratio; NSTEMI = non-ST-elevation myocardial infarction; OR = odds ratio; STEMI = ST-elevation myocardial infarction. * Adjusted HR.
